# Commitments for Ethically Responsible Sourcing, Use, and Reuse of Patient Data in the Digital Age: Cocreation Process

**DOI:** 10.2196/41095

**Published:** 2023-05-05

**Authors:** Sally Okun, Morgan Hanger, Letitia Browne-James, Tara Montgomery, Gary Rafaloff, Johannes JM van Delden

**Affiliations:** 1 Clinical Trials Transformation Initiative Durham, NC United States; 2 Victorious Living Counseling and Consulting, LLC Orlando, FL United States; 3 Civic Health Partners Brooklyn, NY United States; 4 Meridian Investment Partners LLC Westlake, FL United States; 5 Department of Medical Humanities Julius Center for Health Sciences and Primary Care University Medical Center Utrecht, Utrecht University Utrecht Netherlands

**Keywords:** co-creation, patient-centered, health data, ethics, digital age, digital normal, #newdigitalnormal, digital, health, data, artificial intelligence, tool, online, community, communication

## Abstract

**Background:**

Personal information, including health-related data, may be used in ways we did not intend when it was originally shared. However, the organizations that collect these data do not always have the necessary social license to use and share it. Although some technology companies have published principles on the ethical use of artificial intelligence, the foundational issue of what is and is not acceptable to do with data, not just the analytical tools to manage it, has not been fully considered. Furthermore, it is unclear whether input from the public or patients has been included. In 2017, the leadership at a web-based patient research network began to envision a new kind of community compact that laid out what the company believed, how the company should behave, and what it promised both to the individuals who engaged with them and to the community at large. While having already earned a social license from patient members as a trusted data steward with strong privacy, transparency, and openness policies, the company sought to protect and strengthen that social license by creating a socially and ethically responsible data contract. Going beyond regulatory and legislative requirements, this contract considered the ethical use of multiomics and phenotypic data in addition to patient-reported and generated data.

**Objective:**

A multistakeholder working group sought to develop easy-to-understand commitments that established expectations for data stewardship, governance, and accountability from those who seek to collect, use, and share personal data. The working group cocreated a framework that was radically patient-first in its thinking and collaborative in the process of its codevelopment; it reflected the values, ideas, opinions, and perspectives of the cocreators, inclusive of patients and the public.

**Methods:**

Leveraging the conceptual frameworks of cocreation and participatory action research, a mixed methods approach was used that included a landscape analysis, listening sessions, and a 12-question survey. The methodological approaches used by the working group were guided by the combined principles of biomedical ethics and social license and shaped through a collaborative and reflective process with similarities to reflective equilibrium, a method well known in ethics.

**Results:**

*Commitments for the Digital Age* are the output of this work. The six commitments in order of priority are (1) continuous and shared learning; (2) respect and empower individual choice; (3) informed and understood consent; (4) people-first governance; (5) open communication and accountable conduct; and (6) inclusivity, diversity, and equity.

**Conclusions:**

These 6 commitments—and the development process itself—have broad applicability as models for (1) other organizations that rely on digitized data sources from individuals and (2) patients who seek to strengthen operational policies for the ethical and responsible collection, use, and reuse of that data.

## Introduction

### Context

The volume of data generated from digital tools and devices grows unabatedly every day. Although we may acknowledge uncertainty about how the data we generate are collected, used, and shared, often these concerns are offset by the convenience digital devices bring to our daily lives. Yet, these conveniences mask the reality that our data, including health-related data, may be used in ways we did not intend when they were originally shared [1].

Data are a unique commodity of the 21st century. Access to and use of data has proven to be a successful business model for many of the world’s most profitable companies. US regulatory and legislative requirements designed to protect personal information, including health-related data, fall short of ensuring complete control over data access. The General Data Protection Regulation of the European Union, considered to be one of the most restrictive data protection laws in the world, allows for the secondary use of data for research purposes without explicit consent that, based on findings from a German study, was acceptable to a majority of survey respondents [2]. Yet, each piece of data represented in these large data sets begins with individuals who may be unaware of how their data are being used for secondary purposes. In fact, in a small study of companies offering services to patients with cancer, the companies found themselves unaware that they were exposing information about patients they serve through Facebook via cross-site tracking middleware [3]. Additionally, with the advanced technological methodologies available today, reidentification of individuals within large data sets is increasingly possible [4].

In a recent study, researchers seeking to identify factors that influence consumers’ willingness to share their digital data found that privacy concerns shifted with the context of use and reuse but that overall willingness was strongly influenced by underlying concerns about privacy [5]. In other research, empirical evidence related to data sharing revealed that the social license to collect, use, and share data cannot be presumed by organizations and companies that seek it [6]. When National Health Service (England) launched *care.data* to extract data from medical records in 2013, its failure to seek social legitimacy proved negatively consequential [7]. Social license from the public and patients related to data use is conditional and should take into consideration specific expectations that reflect people’s diverse values, needs, and interests [8].

Technology companies seeking to harness the power of large and ever-expanding data sets have developed expertise in rapidly emerging data science methodologies. By 2017, Apple, Google, DeepMind, Microsoft, IBM, and others in the technology sector founded the Partnership on AI (artificial intelligence) with the goal of building a diverse, balanced, and global set of perspectives on AI [9]. Some of these companies went on to publicly announce their own principles on AI [10,11]. These declarations were drafted mainly from the perspective of those who collect, hold, and use vast volumes of data; it is not clear whether input from the public or patients (in the case of health-related data) was considered. In time, both large-scale breaches of individual data and unethical uses of data came into public view, underscoring the broader social implications of insufficient security and opaque data access, use, and reuse policies [12-14].

In contrast, the cocreation process described herein serves multiple stakeholders, including the companies and organization that collect, use, and reuse data and the patients and public from whom data are derived with 6 socially and ethically responsible cocreated commitments.

### Goals

The goal of this project was to develop a radically patient- and public-first set of commitments for the ethical and responsible collection, use, and reuse of data for the digital age. To accomplish this goal, the working group used a participatory research approach guided by cocreation, a collaborative design process in which interaction with consumers plays a central role from start to finish [15].

The work described herein contributes new knowledge to the field of data governance and stewardship derived directly from patients and the public. The core foundational question—*what is and isn’t acceptable to do with data, not just the analytical tools and legal policies to manage it?—*grounded the work of all participants throughout the cocreation process.

The results are 6 commitments that go beyond the minimum data governance standards of security and privacy by prioritizing social legitimacy, accountability, and trust as essential components of ethical and responsible data stewardship.

### Background

In 2017, a multistakeholder working group convened by PatientsLikeMe (PLM), a web-based patient research network [16], was tasked with creating the structure and operational policies for an independent Ethics and Compliance Advisory Board (ECAB) and articulating for patients and the world what good data stewardship should look like [17].

In January 2018, a working group was formed, including the authors of this paper, to cocreate a new kind of community compact to lay out how a company that collects, uses, and reuses shared data ought to behave and what it promises both to the individuals who engage with them and to the community at large. *Commitments for the Digital Age* are the output of this work.

## Methods

### Objective

The objective of the working group was to generate a set of easy-to-understand commitments designed to have an impact in the real world that established expectations for good stewardship, governance, and accountability from those who seek to collect, use, and share personal data. As such, the project was not research-led or driven by publication goals per se. Our methods were intended to maximize the opportunity to deliver a set of real commitments with positive benefits for patients and the public.

To accomplish this objective a mixed methods approach was used including a landscape analysis, listening sessions, and a 12-question survey that embodied the conceptual frameworks of cocreation and participatory action research.

### Recruitment of Cocreators

Invitations to participate in the 1-hour listening sessions were sent via a private electronic message to company employees and to current and alumni members of PLM Team of Advisors (TOA). TOA members are selected annually via an application process to provide insight and perspective on company activities and initiatives. Listening sessions with company employees were held in person. Sessions with TOA were held virtually via a web-based meeting platform.

Invitations to participate in the 12-question survey were sent via a private message to members of the PLM Ambassadors, a larger set of PLM members not selected to join a TOA cohort who agreed to be available to participate in ad hoc activities seeking patient perspective and active involvement. Completion of the survey took approximately 15 minutes.

### Ethical Considerations

Participation in the listening sessions and survey was voluntary, and members were not remunerated for their participation. Independent ethics review was not sought as members of the PLM TOA and Ambassador groups previously agreed to engage in activities seeking their perspective and insight on a variety of topics. The plain language overview of PLM Privacy Policy is accessible on every page of the website. The overview describes how data are used and shared and includes a link to full policy [18].

### Data Collection and Analysis

The qualitative data generated from the listening sessions were documented by 2 members of the working group who together reviewed their notes after each session. Themes were categorized to differentiate those who offered observations about the general tone and intent of the commitments from those who provided specific recommendations for vocabulary changes and other editorial suggestions. A summary of each listening session was discussed with the working group during regularly scheduled meetings, and decisions regarding refinements to the commitments were consensus driven.

Survey data were collected using a proprietary survey tool and stored in a secure database. Two members of the working group reviewed the raw data for survey completeness. The qualitative data were independently reviewed by 2 working group members using the thematic categories previously created for the listening sessions. Graphs and charts representing the quantitative survey responses were created along with a summary of the analysis of the qualitative responses. The working group discussed the survey results and qualitative findings during regularly scheduled meetings, and decisions regarding refinements to the commitments were consensus driven.

### Participatory Research Process

The working group integrated the principles of social license and the conceptual framework of cocreation into the development of the commitments to embody a participatory action research approach [18] defined as follows:

A reflective process that allows for inquiry and discussion as components of the “research.” Often, action research is a collaborative activity among colleagues searching for solutions to everyday, real problems. ([19], p. 6)

The essential components of social license—legitimacy, credibility, and trust—were well established in the web-based patient network. The environment provided a suitable foundation for using the conceptual framework of cocreation. Cocreation is characterized by a continuous and iterative process within which participants play an active role and often use mixed methods for gathering qualitative and quantitative data. Cocreation inspires the generation of unique views and ideas that reflect the collective knowledge, expertise, and perspectives of all involved [20].

As patients, employees, and subject matter experts participated in cocreation, the working group refined the content and structure of the commitments. This process bore similarities to a method well known in ethics—reflective equilibrium—the essence of which is to go back and forth between different sources of empirical and normative information to reach a well-argued position [21].

The methodological approaches used by the working group were guided by the combined principles of biomedical ethics and social license and shaped through a collaborative and reflective process. We sought to anchor both the process of cocreation and the outputs that emerged with the web-based patient research network’s core values of patient-first, transparency, openness, and ensuring trust [22]. These values laid the foundation for 3 key criteria that guided the project’s work. First, the commitments must reflect patient and public participation from start to finish. Second, the commitments must be grounded in ethical principles. Third, the commitments must include the essential components of social license. We also turned to the Council for International Organizations of Medical Sciences (CIOMS) guidelines—notably Guideline 12 that addresses the collection, storage, and use of data in health-related research—as a frame of reference [23].

## Results

### Process

Over 9 months of iteration and input, we developed 28 versions of the commitments document. The first version of the document was inspired by the concept of earning and maintaining social license from those entrusting the company with their personalized data. It also sought to incorporate tenets of health care ethics and focused heavily on autonomy: self-determination, negative autonomy (that people should expect freedom from external interference in one’s own life), and positive autonomy (that people should be able to evolve, develop, and shape their lives according to their own desires and nature).

Subsequent versions benefited from continuous processes of ideation and refinement by the cocreators who included patients, consumers, bioethicists, legal scholars, employees, and company leadership. Trust in the process was an essential component of its success.

**Figure 1:**
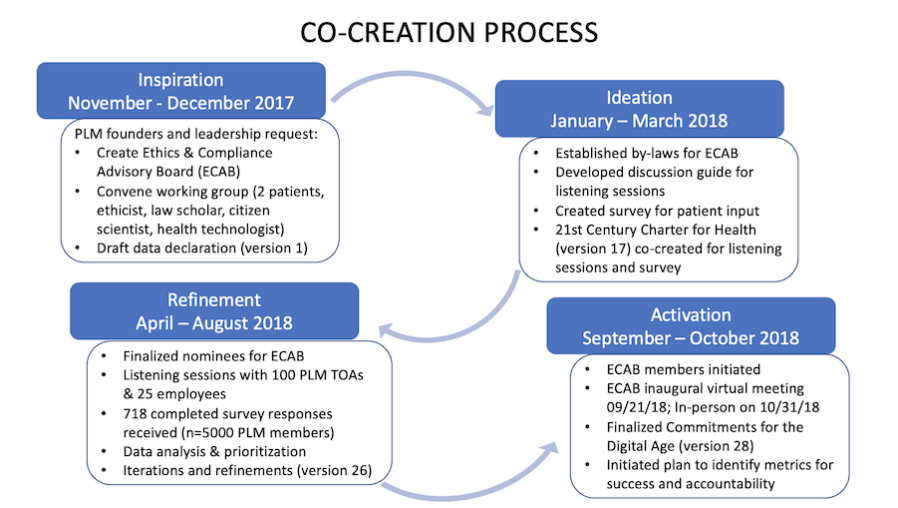
Cocreation process used to develop Commitments for the Digital Age. PLM: PatientsLikeMe; TOA: Team of Advisors.

Figure 1 describes the chronology of activities, events, and outcomes that occurred during each phase of the cocreation process.

### Inspiration Phase

In November 2017, PLM leadership approved a recommendation by the Vice President of Policy and Ethics and an external medical ethicist to establish an independent ethics advisory group. In addition, a founder asked for “a public declaration that states what we believe, how we behave, and based on what we promise what members of PLM can expect when engaging in research with us.” The work on both activities began in December 2017, resulting in the first iteration of the company’s *Community Compact* and an outline for the structure and composition of what came to be called the ECAB. By the end of December, version 4 of the document evolved titled *Our Path for Continuously Earning and Keeping Our Social License*.

### Ideation Phase

A small working group, including 2 patients, a health technologist, a legal scholar, and an open-source data scientist, was formed to help guide the work of establishing the ECAB and cocreating the public declaration of commitments to participants in company research. Between January and March 2018, the group engaged in a modified Delphi process including a series of in-person meetings, phone calls, and rounds of independent document review between discussions. No questionnaire was used, but a single facilitator led and documented each of the discussions. During this time, the declaration evolved from focusing equally on beliefs, values, and commitments, to focusing more intentionally on clear, robust commitments to which a company that collects, uses, and reuses patient data could be held accountable. By March 2018, version 17 of the declaration titled *21st Century Charter for Health* was ready for refinement with a broader audience.

### Refinement Phase

The working group solicited input on the evolving document from 3 sources during this phase. All participants described below received the *21st Century Charter for Health* document before engaging in the activity. The 3 sources of data included the following:

- One-hour listening sessions with PLM TOAs, a patient-only panel that helps bring the patient voice to the forefront of health care (n=100)
- One-hour listening sessions with PLM employees (n=25)
- Twelve-question survey deployed to PLM Ambassadors [24], a larger set of PLM members interested in participating in an advisory capacity (n=5000). A total of 718 completed survey responses were received.

The qualitative listening sessions provided an opportunity for participants to share their perspectives on a range of topics related to the content of the *21st Century Charter for Health*. Many people reiterated the importance of the charter as a foundational document. Others noted the importance of individuals knowing and choosing to participate in research generally, as well as in specific studies involving their personal data and biological specimens. They valued the ability to opt in and opt out, especially as their health status evolved and changed over time. Many expressed interest in better understanding the mechanism by which the company would hold itself accountable to the tenets covered in the charter. Some participants indicated the need for reassurance around the authenticity of the charter and its meaningful implementation in the real world. A breach of the charter would represent not only a breach of policy and ethical guidelines but also a violation of the company’s core identity and a betrayal of its promise to the patient community.

Respondents to the quantitative survey were asked to select the single most important commitment among the 6 presented (Table 1). Two priorities emerged: “continuous learning environment” (33%) and “respect for autonomy” (26%), with the other 4 each receiving 12% or less. When prompted, 39% reported that “protecting my data and privacy” was the most important topic area, followed by 24% reporting “new technology development and their implications to me.” Generally, respondents gravitated toward commitments related to preserving some control over how their data were used and ensuring that, to whatever extent possible, their data provided meaning through new insights and shared learning. This led the working group to reprioritize the commitments with “continuous and shared learning” listed first moving forward.

**Table 1:** Most and least important priorities of patient respondents to the survey (N=718).

Commitment	Priority, n (%)
Continuous learning environment	235 (33)
Respect for autonomy	186 (26)
Accountable governance	85 (12)
Open communication	81 (11)
Value for people	64 (9)
Safe and responsible conduct	61 (8)
I prefer to skip	6 (1)

At the end of the survey, respondents were provided with a free text box for comments (Table 2). There were 191 free-text responses, many of which were high level or directional. The preponderance of comments (“positive feedback”) noted the value of this kind of document and solicitation of patient input, for example, “this is the right time to make these things clear” and “I think it covers a lot of concerns people may have” (43%). Another 18% were broadly related to text edits or structural considerations, for example, “it’s too long” and “sometimes I got lost reading it.” Nine percent of comments shared something personal to the respondents or conveyed something about their survey experience, for example, “[my condition] is horrid,” or “I skipped one question because [the options] are all important....” Ten percent were related to PLM services or functionality that are unrelated to the topics covered in the survey.

Regarding specific, substantive themes, 15% were related to privacy and security, though some of these were noting recent data breeches or acknowledging related trade-offs, for example, “I think there is a point when the potential value of data outweighs the need for protection,” rather than merely expressing concern. Nine percent of the comments were related to selling data, some opposing it in general and some requesting additional clarity about company practices, for example, “Do you sell the data to researchers… Do you give data to pharmaceutical companies as they do research?” Eight percent expressed concern about the accessibility of these commitments, for example, “I know if I wasn’t taking the survey, I’m not sure I would have read it as thoroughly as I did.” Finally, 8% noted the importance of accountability, specifically the company leadership’s willingness to follow through on the commitments, for example, “Who has written this document? How well do they represent the executives?” and “It sounds pretty good, reads very positive. Of course, it’s just a bunch of words that don't do anything themselves.” This healthy skepticism underscored an activity that the ECAB was already considering, namely the creation of success metrics to evaluate the company’s performance over time.

**Table 2:** Free-text survey responses underscore importance and concerns of patients about commitments (N=191).

Comment	Value n (%)
Positive feedback	82 (43)
Edits and structure	35 (18)
Privacy and security	28 (15)
Feedback to PatientsLikeMe	19 (10)
Personal comment	17 (9)
Selling data	17 (9)
Leadership intentions	16 (8)
Access and understanding	13 (7)

After completing an analysis of the qualitative and quantitative data, the working group refined the 6 commitments in the charter and reprioritized the order to reflect respondent input.

In addition to the iterative work on the charter, the process of establishing the ECAB continued with identifying and vetting nominees. The newly selected ECAB members received a briefing packet that included the cocreated version 26 titled *21st Century Charter for Health.*

### Activation Phase

The newly established ECAB met virtually for the first time in September 2018. The 9 members, 5 of whom were patients and consumer representatives, discussed the commitments document and provided feedback on language, tone, and title. The ECAB finalized the document now titled *Commitments for the Digital Age* (Textbox 1). The members agreed that the next phase of the cocreation process would seek to identify metrics of what success looks like for each of the 6 commitments.

**Commitments for the digital age.**

**Continuous and shared learning**

- We are continuously developing and advancing a robust and dynamic knowledge repository as a continuous learning network, where meaningful data and insights are shared to support decision-making, and where effective tools to act on those decisions help people thrive on their own terms.

**Respect and empower individual choice**

- We respect each person’s autonomy and the relationships that support it. In our view, autonomy also means each person controls their data and its use.
- We enable individuals to choose the services that fit them, to choose the level of privacy they want, and to partner in research that interests them.
- Individuals decide what they want to contribute to and to be informed (or not) about individual or collective results.

**Informed and understood consent**

- We believe that informed consent is much more than a signed form.
- It is an adaptive mutual process in which individuals are given timely information, they need to understand the choices available to them, and they are free to act on those choices without interference.

**People-first governance**

- Each piece of data is a moment in real life that must be respected.
- We have a profound responsibility to protect that data to make sure that how, when, and why they are accessed or used, and by whom, aligns with the personal interests of individuals, the collective norms of their community, and the laws and regulations of the country they call home.

**Open communication and accountable conduct**

- We will continually encourage feedback, and we will keep ourselves honest about providing opportunities to do so.
- We also know we have a responsibility to share what we learn, our successes, and our mistakes proactively with individuals and with the world at large.

**Inclusivity, diversity, and equity**

- We acknowledge that like most digital and web-based platforms we must have greater inclusivity and diversity in our environment, and that this must be practiced, not preached.
- We seek to understand diverse perspectives and believe that everyone benefits when we combine the power of our collective human voices with advances in biomedical and biological research and the dynamic technologies with which we interact in our daily lives.

## Discussion

### Principal Findings

Through a continuous and intense process of cocreation spanning 9 months, 6 commitments for the digital age were developed to provide ethically responsible guidance for shared power and governance between a company that collects, uses, and reuses data and the community from whom data are sourced.

The 6 commitments in order of priority as determined through the cocreation process are (1) continuous and shared learning; (2) respect and empower individual choice; (3) informed and understood consent; (4) people-first governance; (5) open communication and accountable conduct; and (6) inclusivity, diversity, and equity. In these commitments, the ethical norms of privacy and security are clearly visible. At the same time, there is a strong commitment to respect autonomy and to help people thrive on their own terms by starting from the diverse lived experience of each individual.

The final version of *Commitments for the Digital Age* embraces the inherent tension that exists for patients in a digitally informed era. Patients can understand and appreciate the value to themselves and society of using data for good to solve complex problems related to health and illness. Yet, they also recognize that companies that collect, store, use, and reuse their data may lack the ethical maturity needed to act responsibly.

The processes undertaken represent a collaborative experiment to create an advisory structure and set of expectations through which a business and a self-selected community can successfully partner to navigate new and uncertain ethical spaces related to privacy, autonomy, equity, and the potential individual impact of participating in novel research endeavors. There was an additional relational effect of going through this process with a company’s employees and constituents together. In cocreation, those involved started to experience the philosophical benefit of cocreation itself, a process that was more than creating a document. Over the iterations, the commitments became less about a company’s beliefs and more about a company’s commitment to ethically responsible conduct. In essence, *Commitments for the Digital Age* embody principles and behaviors we should all expect from companies that collect, use, and reuse our data.

### Comparison With Other Work

In comparing our work with other literature and conceptual frameworks, we noted interesting differences that illuminate the dearth of patient and public participation in data governance and stewardship. A systematic review of literature published between 2001 and 2009 on the state of data governance in companies and corporations prompted the paper’s authors to develop a new conceptual framework for data governance and to recommend 5 promising fields for future research [25]. The omission of public participation in the reviewed publications is noteworthy. The absence of the public in the new multidimensional conceptual framework and in recommendations for future research on data governance indicates a critical gap in knowledge and practice that our cocreated commitments are well positioned to fill. A nationally representative survey of US adults conducted by the Association of American Medical Colleges Center for Health Justice in January 2022 found that the tension related to the public’s willingness to share personal health data for the common good persists reinforcing the timeliness of the cocreated *Commitments for the Digital Age* [26].

Widely used ethical frameworks that are relevant to health data such as the World Health Organization/CIOMS principles, the Belmont report, and the Taipei principles of the World Medical Association have ethics committees as their prime addressee [21,27,28]. In the commitments, the members and other publics are the prime addressees, which is appropriate as the commitments’ aim is to protect the social license the company earned from its members. Also, the more recent reports of CIOMS and Taipei restrict themselves to a meta-level primarily, as they call for the creation of standard operating procedures on several specific issues, such as the return of results of research. However, these documents provide less guidance around the actual content of these standard operating procedures.

The cocreated commitments, while general in nature, do offer actionable guidance when considered in specific use cases. DATAGov, a project that seeks to coproduce a “model for involving patients and the public in decision-making processes about the use and sharing of health data for rare diseases” is a prime example [29]. The project, which employed similar participatory research methods, could strengthen social legitimacy and accountability by making the *Commitments for the Digital Age* an integral public facing component of its coproduction processes on data use and sharing for care and research of rare diseases.

The timeliness of our work and relevance beyond the original intent are made obvious in a recent Harvard Business School series titled *Leading in the Digital Age* [30]. The series documents insights of senior executives from companies around the world on digital transformation and digital maturity. A passage from the first article of the series published in January 2022 aptly captures the essence of the *Commitments for the Digital Age* as a clear and relevant moral compass for any company seeking to develop its digital maturity [31]:

Leaders of digitally mature organizations align their employees around a shared purpose that puts ethical decision-making on behalf of stakeholders at the center. These companies earn the right to collect and use employees’ and customers’ data, for example, by being transparent about their intentions and relevant processes. When they use that data, they actively ensure they are abiding by the expectations they set when they gather it. Organizations want to get to the point where customers want to share their personal information because they trust they will benefit from its use. Building this trust needs to be a multi-pronged effort embraced by all in the company—not just policed by those in compliance roles. (p. 5)

### Limitations

It is important to note limitations in the generalizability of our work, in terms of both the process and the commitments. First, the web-based patient research network had an active and engaged member base that was available to participate. There was already a baseline understanding about some of the principles we engaged them on. Many companies may not have access to this kind of population. Second, and related, is an inherent bias in our sample as members of the PLM network, by definition, are people participating in a web-based data sharing platform. Furthermore, survey participants may represent a more engaged, equipped, and empowered subset of the company’s patient membership. Third, the company had an executive (SO) entirely dedicated to ethics and policy who was able to dedicate significant time to the logistics and continuous learning this project required; such a resource may not be available within similar organizations.

Another important limitation is that because of a change in the ownership of the company, the commitments were not codified upon their completion. Therefore, we cannot comment on the challenges of adoption, implementation, or maintenance over time, or the related accountability mechanisms. Full realization of these commitments is undoubtedly a rigorous and continuous undertaking that requires ongoing socialization, reflection, measurement, and adaptation.

### Conclusions

A working group came together to cocreate with a web-based patient community a public declaration of commitments for ethically responsible behaviors for data collection, use, and reuse to which a company could be held accountable by those with whom it engaged in the conduct of its business. The declaration would respect current ethical, legal, and societal standards for responsible collection, use, and reuse of patient data in the 21st century era, and it would be adaptable to ever-evolving technological capabilities.

We believe that these commitments have broad applicability and call upon health systems, clinical organizations and providers, technology companies, software developers, data scientists, researchers, and others who rely on data sourced from individuals to use our work as a model for making similar commitments. In this digital era, the public deserves trustworthy stewards of their data who willingly integrate and publicly share the ethical and social commitments to which they can be held accountable.

## Abbreviations

AI: artificial intelligence
CIOMS: Council for International Organizations of Medical Sciences
ECAB: Ethics and Compliance Advisory Board
PLM: PatientsLikeMe
TOA: Team of Advisors

## Data Availability

The data sets generated and analyzed during this study are not publicly available as they contain information that could compromise research participant privacy and consent.

## References

[1] Hinds J, Williams EJ, Joinson AN (2020). “It wouldn't happen to me”: privacy concerns and perspectives following the Cambridge analytica scandal. Int J Hum Comput Stud.

[2] Richter G, Borzikowsky C, Lieb W, Schreiber S, Krawczak M, Buyx A (2019). Patient views on research use of clinical data without consent: legal, but also acceptable?. Eur J Hum Genet.

[3] Downing A, Perakslis E (2022). Health advertising on Facebook: privacy and policy considerations. Patterns (N Y).

[4] Farzanehfar A, Houssiau F, de Montjoye YA (2021). The risk of re-identification remains high even in country-scale location datasets. Patterns.

[5] Grande D, Mitra N, Iyengar R, Merchant RM, Asch DA, Sharma M, Cannuscio CC (2022). Consumer willingness to share personal digital information for health-related uses. JAMA Netw Open.

[6] Muller SHA, Kalkman S, van Thiel GJMW, Mostert M, van Delden JJM (2021). The social licence for data-intensive health research: towards co-creation, public value and trust. BMC Med Ethics.

[7] Carter P, Laurie GT, Dixon-Woods M (2015). The social licence for research: why care.data ran into trouble. J Med Ethics.

[8] Kalkman S, van Delden J, Banerjee A, Tyl B, Mostert M, van Thiel G (2022). Patients' and public views and attitudes towards the sharing of health data for research: a narrative review of the empirical evidence. J Med Ethics.

[9] (2021). Partnership on AI.

[10] Responsible AI. Microsoft.

[11] Pichai S (2018). AI at Google: our principles. Google.

[12] Isaak J, Hanna MJ (2018). User data privacy: Facebook, Cambridge analytica, and privacy protection. Computer.

[13] Barnett I, Torous J (2019). Ethics, transparency, and public health at the intersection of innovation and Facebook's suicide prevention efforts. Ann Intern Med.

[14] Rességuier A, Rodrigues R (2020). AI ethics should not remain toothless! A call to bring back the teeth of ethics. Big Data Soc.

[15] Rezaei Aghdam A, Watson J, Cliff C, Miah SJ (2020). Improving the theoretical understanding toward patient-driven health care innovation through online value cocreation: systematic review. J Med Internet Res.

[16] PatientsLikeMe.

[17] Okun S, Wicks P (2018). DigitalMe: a journey towards personalized health and thriving. BioMed Eng OnLine.

[18] Privacy policy. PatientsLikeMe.

[19] Eckhardt J, Kaletka C, Krüger D, Maldonado-Mariscal K, Schulz AC (2021). Ecosystems of co-creation. Front Sociol.

[20] Sendall MC, McCosker LK, Brodie A, Hill M, Crane P (2018). Participatory action research, mixed methods, and research teams: learning from philosophically juxtaposed methodologies for optimal research outcomes. BMC Med Res Methodol.

[21] Ferrance E (2000). Action research. Education Alliance.

[22] Rawls J, Cohen M (2018). A theory of justice. Princeton Readings in Political Thought: Essential Texts from Plato to Populism.

[23] About. PatientsLikeMe.

[24] Council for International Organizations of Medical Sciences (CIOMS), World Health Organization (2016). 2016 International ethical guidelines for health-related research involving humans. 4th ed. Council for International Organizations of Medical Sciences (CIOMS).

[25] Bradley M, Braverman J, Harrington M, Wicks P (2016). Patients' motivations and interest in research: characteristics of volunteers for patient-led projects on PatientsLikeMe. Res Involv Engagem.

[26] For the common good: data, trust, and community health. AAMC Center For Health Justice.

[27] Abraham R, Schneider J, vom Brocke J (2019). Data governance: a conceptual framework, structured review, and research agenda. Int J Inf Manage.

[28] National Commission for the Protection of Human Subjects of Biomedical and Behavioral Research (1978). The Belmont Report: Ethical Principles and Guidelines for the Protection of Human Subjects of Research.

[29] (2002). Declaration of Taipei on ethical considerations regarding health databases and biobanks. The World Medical Association.

[30] de Freitas C, Amorim M, Machado H, Leão Teles E, Baptista MJ, Renedo A, Provoost V, Silva S (2021). Public and patient involvement in health data governance (DATAGov): protocol of a people-centred, mixed-methods study on data use and sharing for rare diseases care and research. BMJ Open.

[31] Hill LA, Le Cam A, Menon S, Tedards E (2022). Where can digital transformation take you? Insights from 1,700 leaders. Harvard Business School Working Knowledge.

